# An investigation into the sex dependence of post‐reperfusion cardiac mitochondrial function and redox balance in chronically stressed rats

**DOI:** 10.14814/phy2.70185

**Published:** 2025-02-25

**Authors:** Megan Cairns, Jasmine Andrews, Caitlin Odendaal, Cassidy O'Brien, Erna Marais, Gerald Maarman, Balindiwe Sishi, Danzil Joseph, Fanie Rautenbach, Jeanine L. Marnewick, M. Faadiel Essop

**Affiliations:** ^1^ Centre for Cardio‐Metabolic Research in Africa (CARMA), Division of Medical Physiology, Department of Biomedical Sciences, Faculty of Medicine and Health Sciences Stellenbosch University Cape Town South Africa; ^2^ Center for Cardio‐Metabolic Research in Africa (CARMA), Department of Physiological Sciences Stellenbosch University Stellenbosch South Africa; ^3^ Oxidative Stress Research Centre, Faculty of Health and Wellness Sciences Institute of Biomedical and Microbial Biotechnology, Cape Peninsula University of Technology Cape Town South Africa

**Keywords:** chronic stress, heart, ischemia–reperfusion, mitochondrial respiration, sex‐based differences

## Abstract

Although mitochondrial alterations are implicated in cardiac pathologies, sex‐specific changes following chronic stress and ischemia–reperfusion injury are poorly characterized. Male and female Wistar rats underwent chronic restraint stress (CRS) for 4 weeks versus controls, whereafter *ex vivo* hearts were subjected to regional ischemia and reperfusion. Post‐reperfusion hearts were dissected into ischemia‐reperfused and non‐ischemic regions with high‐resolution mitochondrial respirometry, and oxidative stress assays performed. CRS males displayed increased routine and fatty acid β‐oxidation respiration in non‐ischemic tissues but lowered ETF‐linked LEAK contributions to overall electron transfer system capacity ratios in ischemia‐reperfused regions versus controls. CRS males exhibited lowered superoxide dismutase activity and increased lipid peroxidation in well‐perfused regions versus controls. Female CRS hearts showed attenuated ETF‐linked LEAK respiration and increased lipid peroxidation versus controls in non‐ischemic tissue but a lowered RE ratio (measure of mitochondrial coupling) with ischemia–reperfusion. Our findings highlight the heart's sexually dimorphic response to chronic stress and ischemic injury, with female hearts showing oxidative damage in non‐ischemic tissues together with relatively intact mitochondrial function in ischemia‐reperfused tissues.

## 1 INTRODUCTION

Ischemic heart disease is the leading cause of cardiovascular diseases globally, contributing to over 50% and 45% of disability‐adjusted life years in men and women, respectively (Aggarwal et al., 2018; Dattani et al., 2023; World Health Organization, 2024). Growing clinical evidence highlights a bidirectional relationship between ischemic heart disease and psychosocial stress. For example, individuals with anxiety, depression, or post‐traumatic stress disorder display a higher risk for major adverse cardiac events (Abbasi et al., 2016; Edmondson et al., 2011). Conversely, patients diagnosed with cardiometabolic diseases are more susceptible to developing psychological stress (Ebrahimi et al., 2021; Jee et al., 2019; Rutledge et al., 2014; Sumner et al., 2015; Xian et al., 2010). While there are limited pre‐COVID‐19 pandemic studies investigating the direct prevalence of chronic stress, a wealth of research is now available regarding the prevalence of psychological conditions like post‐traumatic stress disorder, depression, and anxiety (Al Omari et al., 2020; Iasevoli et al., 2021; Xiong et al., 2020). Such data serves as a useful proxy for tracking the global rise in chronic stress. These psychological conditions are known to alter physiological pathways—particularly those involved in cardiovascular responsiveness—and thereby contributing to the development of cardiovascular diseases such as stroke and heart failure (Hou et al., 2021; Mousavi et al., 2019; Ndzie Noah et al., 2023; Zoladz et al., 2019).

However, a critical knowledge gap exists regarding the sex‐specific pathophysiology of chronic stress and its downstream effects on cardiovascular health. Moreover, preclinical studies investigating chronic stress predominantly rely on male animal models, neglecting potential sex‐based differences and limiting a holistic understanding of the problem (Lopez & Bagot, 2021). This is concerning as males and females exhibit distinct stress responses, together with different clinical presentations of adverse cardiac conditions (Aggarwal et al., 2018; Pravda et al., 2021). The elucidation of sex‐specific mechanisms is therefore crucial to enhance our understanding of increased major adverse cardiac events in stressed individuals, particularly when considering ischemia–reperfusion injury.

Although mitochondrial respiratory functionality is crucial in terms of understanding such cardiac pathology, relatively limited (sex‐dependent) information exists regarding stress‐related ischemia–reperfusion injury. Mitochondria play a pivotal role in cardiovascular health by ensuring a steady supply of intracellular ATP, vital for optimal cardiac function (Scott et al., 2022). While mitochondria generate physiological levels of reactive oxygen species (ROS), enhanced production can trigger adaptive mechanisms such as mitochondrial fusion or fission events in an attempt to counteract damaging effects and thereby sustain cardiac function (Tham et al., 2015). However, if such attempts fail and mitochondrial ATP levels remain relatively low, apoptosis and cardiac dysfunction can result (Dorn & Kitsis, 2015; Kubli & Gustafsson, 2012; Tham et al., 2015). While proteomic studies revealed differential pathway responses in infarcted and unaffected cardiac tissues during regional ischemia, the mitochondrial respiratory responses remain relatively unknown (Mezger et al., 2022).

In light of this, the current preclinical study aimed to address this knowledge gap by investigating sex‐based differences in mitochondrial function and redox balance following chronic stress exposure and subsequent ischemia–reperfusion injury. This approach was adopted to gain valuable insights into the sex‐specific mechanisms that contribute to cardiovascular complications in response to chronic stress.

## 2 MATERIALS AND METHODS

### 2.1 Ethics and CRS model

Ethical clearance was obtained from the Animal Ethics Committee of Stellenbosch University, South Africa (ACU‐19400). The animals used in this study were handled by qualified personnel and treated according to the Guidelines for the Care and Use of Laboratory Animals of the National Academy of Science (NIH publication No. 85–23, revised 1996). All rats were fed a standard rodent chow (Rodent Breeder Customized Laboratory Animal Diet; #LAB/RB2005; NutritionHUB (Pty) Ltd.) with *ad libitum* access to water, except during experimental interventions. Housing conditions included a stable temperature and humidity of 22°C and 50%, respectively.

This study constitutes a molecular‐based continuation on stored samples collected during a larger investigation conducted by our laboratory where circulating biochemical and *ex vivo* heart functional parameters were assessed (Cairns et al., 2024). Seven‐week‐old male and female Wistar rats were randomly divided into “control” and “chronic restraint stress (CRS)” groups. At 10 weeks of age, rats in the stress group were subjected to daily restraint within Perspex cages (6 cm × 7 cm × 18 cm) for 4 weeks whereas the control group also received daily handling for a maximum of 15 min per cage to prevent potential handling stress during weekly bodyweight measurements and/or behavioral tests, while also mirroring the handling experienced by the stressed animals (Firmino et al., 2019).

Behavioral tests and hormone analyses were performed to validate the successful induction of a chronic stress phenotype as described by us (Cairns et al., 2024). Briefly, behavioral tests (tail flick test and elevated plus maze [EPM]) aimed to evaluate the presence of depression or anxiety‐like behavior, whereas circulating hormones and neurotrophic factors (i.e., brain‐derived neurotrophic factor [BDNF]), particularly those involved in the HPA axis (i.e., corticosterone and adrenocorticotropic hormone [ACTH]), were evaluated via ELISA kits (E‐EL‐0160; E‐EL‐R0048; and E‐EL‐R1235; ElabScience) in stored plasma samples collected 48 hours post‐CRS protocol cessation (between 0800 and 0930) from the jugular vein (Ibironke & Mordi, 2011; Komada et al., 2008; Rorabaugh et al., 2015). Serum 17β‐estradiol (E‐OSEL‐R0001; ElabScience), as well as progesterone and testosterone, were determined (using HPLC) in samples collected from trunk blood after euthanasia (Cairns et al., 2024).

### 2.2 Ex vivo heart perfusions and sample collection

The heart samples utilized for this study were collected following regional ischemia and at the end of the reperfusion stage as previously described by us (Cairns et al., 2024). In short, this protocol included hearts being excised from the chest cavity under surgical‐level anesthesia following a weight‐appropriate injection of sodium pentobarbitone (100 mg/kg; *i.p*.) and immediately arrested in ice‐cold Krebs–Henseleit bicarbonate buffer (119 mM NaCl; 25 mM NaHCO_3_; 4.75 mM KCl; 1.2 mM KH_2_SO_4_; 0.6 mM MgSO_4_.7H_2_O; 0.6 mM Na_2_SO_4_; 1.25 mM CaCl_2_.H_2_O, 10 mM D‐glucose). The hearts were mounted on an *ex vivo* perfusion rig and cannulated for antegrade and retrograde perfusions, with oxygenated Krebs–Henseleit buffer (95% O_2_, 5% CO_2_) serving as the perfusate (Smit et al., 2022). During regional ischemia, the left anterior descending coronary artery (LAD) was ligated with a silk surgical suture for 25 min (Van Vuuren et al., 2016). At the release of the silk suture, the heart began 2 h of reperfusion (10 min stabilization, 20 min working mode, 90 min retrograde perfusion).

After completion of the two‐hour reperfusion period, the LAD was re‐occluded, and 5% (^w^/_v_) Evans blue dye (EBD) manually infused into the non‐LAD‐supplied regions: right ventricle, posterior left ventricle, and remaining left ventricular walls (collateral perfusion dependent) via the aortic cannula during retrograde perfusion (Ogobuiro et al., 2024). We adopted a single EBD stain approach for this particular study as this strategy allowed for downstream techniques such as Western blotting and proteomics, which are not compatible with the tissue processing required for dual staining with triphenyl tetrazolium chloride (which is primarily suited for histological analysis). Hearts were removed from the perfusion rig by performing a transaxial cut inferior to the atria and cut from the apex towards the knot (transverse). Hearts were subsequently dissected into two fractions, that is, the dyed (non‐ischemic tissue) and non‐dyed (ischemia‐reperfused tissue) regions, thereafter snap frozen in liquid nitrogen, and stored at −80°C for future use. Final sample sizes due to perfusion exclusion criteria (e.g., ischemic preconditioning or technical confounding factors) were as follows: male (control = 6; CRS = 5) and female (control = 5; CRS = 7).

### 2.3 Mitochondrial respiration

All experiments were conducted using the SUIT‐005_O2_pfi_D011 protocol on an Oroboros® O2k‐fluorespirometer (Oroboros Instruments, Innsbruck, Austria). Prior to each experiment, air and fluorescent calibrations were performed with the former at air saturation (37°C) and the latter generated by following standard protocols for both hydrogen peroxide and mitochondrial membrane potential. For hydrogen peroxide determination, a calibration curve was generated by incrementally adding 0.1 μM hydrogen peroxide to the 10 μM Amplex™ UltraRed (A36006; Sigma‐Aldrich) fluorescent probe and measuring the corresponding fluorescence intensity (Komlódi et al., 2021). To calibrate for mitochondrial membrane potential, a standard protocol was followed whereby a 1 mM stock solution of tetramethylrhodamine and methyl ester (TMRM; T5428; Sigma‐Aldrich) was titrated stepwise to reach a final range of 0.5–2 μM. The fluorescence intensity of each probe was measured using the O2K's fluorometer, and calibration curves constructed by plotting fluorescence intensity against concentration. Once oxygenated, 10 μM Amplex™ UltraRed (A36006; Sigma‐Aldrich) and tetramethylrhodamine and methyl ester (TMRM; T5428; Sigma‐Aldrich) were added to chambers A and B to measure hydrogen peroxide production and mitochondrial membrane potential, respectively. Amplex™ UltraRed required preceding titrations of 1 U/mL horseradish peroxidase (SRE0082; Sigma‐Aldrich) and 15 μM DTPA (D1133; Sigma‐Aldrich), and in order to produce resorufin, a red fluorescent compound able to be monitored by the fluorescent probes (Fluorescence‐Sensor Green equipped with Filter Set AmR; Ex = 525 nm; Em = ~600; Oroboros Instruments).

We employed frozen heart tissues that were collected post‐reperfusion. Here, ~4 mg of heart tissue was permeabilized in a 50 μg/mL digitonin (D5628; Sigma‐Aldrich)‐MiR05 (0.5 mM ethylene glycol‐bis (2‐aminoethylether)‐N,N,N′,N‐tetraacetic acid [EGTA; E3889; Sigma‐Aldrich], 3 mM magnesium chloride [MgCl2; M8266; Sigma‐Aldrich], 60 mM lactobionic acid [L2398; Sigma‐Aldrich], 20 mM taurine [T0625; Sigma‐Aldrich], 10 mM potassium phosphate monobasic [KH2PO4; P0662; Sigma‐Aldrich], 20 mM 4‐(2‐Hydroxyethyl)piperazine‐1‐ethanesulfonic acid, N‐(2‐Hydroxyethyl)piperazine‐N′‐(2‐ethanesulfonic acid) [HEPES; H3375; Sigma‐Aldrich], 110 mM sucrose [573,113; Sigma‐Aldrich], and 1 g/L essential fatty acid‐free BSA [03117057001; Sigma‐Aldrich]; pH = 7.4) respiratory medium for 20 min on ice after mechanically separating the muscle fibers. Due to the dissection of both dyed and undyed tissue from cross‐sectional heart sections, precise ventricular origin identification for HRR analysis was not feasible. After samples were added to the chambers, hyperoxic conditions were induced and maintained between ~300 and 600 μM throughout the experiment.

Routine respiration was recorded after oxygen flux stabilized (~10 min). Subsequently, glycolytic and fatty acid oxidative substrates were titrated into the chambers according to the standard protocol. Octanoyl (0.5 mM; O6206; Sigma‐Aldrich) and malate (2 mM; M1000; Sigma‐Aldrich) induced electron transfer flavoprotein (ETF)‐linked LEAK respiration, and the addition of ADP (7.5 mM; 117,105; Sigma‐Aldrich) assessed β‐oxidation‐linked oxidative phosphorylation (OXPHOS). Cytochrome c (10 μM; C7752; Sigma‐Aldrich) determined mitochondrial membrane integrity. Pyruvate (5 mM; P8574; Sigma‐Aldrich) and succinate (10 mM; S2378; Sigma‐Aldrich) assessed complex I‐ and complex II‐mediated OXPHOS. Multiple 1 μL titrations (0.5 μM increments) of carbonyl cyanide m‐chlorophenylhydrazone uncoupler (CCCP; C2759; Sigma‐Aldrich) were employed to assess maximal electron transfer system (ETS) capacity. The titrations continued until maximal respiration was achieved. Rotenone (0.5 μM; R8875; Sigma‐Aldrich) and antimycin A (2.5 μM; A8674; Sigma‐Aldrich) measured complex II contribution to ETS and residual oxygen consumption (ROX), respectively. Ascorbate (2 mM; A7631; Sigma‐Aldrich) and tetramethyl‐phenylenediamine (0.5 mM; T3134; Sigma‐Aldrich), followed by sodium azide (200 mM; S2002; Sigma‐Aldrich), assessed complex IV activity. All respiratory states, hydrogen peroxide levels, and membrane potential were corrected for baseline state (ROX) and normalized to sample weight (Gnaiger, 2020). All data were analyzed with DatLab 7.4 (Oroboros Instruments, Innsbruck, Austria). Refer to supplemental table  for a fully detailed titration protocol and Figures S1 and S2 for representative oxygraphs.

Several ratios were calculated utilizing the complex or pathway‐specific oxygen consumption relative to total ETS capacity, namely: total PE (β‐oxidation‐linked OXPHOS + complex II activity/ETS); complex II/ETS (CII/ETS); oxidative phosphorylation coupling efficiency [OPCE] (ETF‐linked LEAK respiration/ETS); and RE (ROX/ETS) to determine mitochondrial coupling in the frozen samples.

### 2.4 ATP determination

Total cardiac ATP levels were determined via an ATP chemiluminescence assay kit (E‐BC‐F002‐M) purchased from ElabScience®. Briefly, 30 mg of frozen heart tissue (ischemic and non‐ischemic zones) pulverized under liquid nitrogen was combined in a 1:9 ratio of cold extraction solution (provided in the assay kit) and homogenized utilizing a bullet blender (4200 rpm; linear speed = 6.90 m/s; six cycles of 1.5:2‐min run: rest intervals) at 4°C. Thereafter, samples were incubated in a boiling water bath for 2 min and centrifuged at 10,000 ***g*** for 10 min. Both samples and standards in the appropriate dilutions were loaded into black 96‐well plates according to kit instructions, mixed with the enzyme working solution in the wells, and read immediately (FLUOstar Omega Multimode Microplate Reader, 415–1364, BMG Labtech, Offenburg, Germany). Absolute sample ATP levels were determined by interpolation of fluorescence values into the linear standard curve. Final concentration values were expressed as μmol/L.

### 2.5 Oxidative stress

Ischemia‐reperfused and non‐ischemic heart tissue samples (50 mg) were pulverized under liquid nitrogen and combined in a 1:9 ratio with 1 M PBS (P4417; Sigma‐Aldrich). To ensure complete subcellular disruption, 1.6 mm stainless steel beads (1 bead: 100 μL lysis buffer) were added to the sample and further homogenized utilizing a bullet blender (4200 rpm; linear speed = 6.90 m/s; six cycles of 1.5: 2‐min run: rest intervals) before centrifugation at 12,080 ***g*** for 20 min (4°C). Protein concentration was determined via the Bradford protein assay and aliquots set aside at −80°C. Various measurements of enzymatic (superoxide dismutase [SOD] and catalase [CAT]) and non‐enzymatic antioxidant capability (ferric reducing ability of plasma [FRAP]), as well as induced oxidative lipid damage (measured as thiobarbituric acid reactive substance [TBARS]), were performed as described previously with each sample loaded in triplicate to reduce technical variability (Geddie et al., 2023). Absorbance readings for assays were measured using a spectrophotometer (Thermo Fisher Scientific, Boston, MA, USA) and the associated data acquisition software (SoftMax® Pro Version 7.0, Molecular Devices, CA, USA).

For SOD activity, samples were combined in a 1:1.25:14 ratio with 6‐OHDA (1.6 mM; 1,002,267,728; Aldrich Chemistry) and a chelating agent (0.1 M diethylenetriaminepentaacetic acid; 1.08390; Sigma‐Aldrich) in a clear 96‐well plate. The absorbance was monitored for 5 min at 490 nm, and final SOD activity expressed as U/mg protein. For CAT activity, heart tissue supernatant, PBS, and 30% (^v^/_v_) H_2_O_2_ (10,366; BDH) were added in a 1:17:5 ratio to a clear 96‐well UV plate. The plate was read at 240 nm for 5 min to monitor the enzymatic breakdown of H_2_O_2_, and the final CAT activity was expressed as U/mg protein. For antioxidant capacity, samples were loaded into a clear, flat‐bottom 96‐well plate in a 1:30 ratio with FRAP reagent (Fe^3+^ chloride hexahydrate [101,048,258; Sigma‐Aldrich], L‐ascorbic acid [A5960; Sigma‐Aldrich], sodium acetate [S8750; Sigma‐Aldrich], 2,4,6‐tri[2‐pyridyl]‐s‐triazine [Sigma‐Aldrich]) and left to incubate at room temperature for ≥30 min (Kubli & Gustafsson, 2012). Once sufficient color development occurred, the plates were read at 593 nm with sample values interpolated into the ferrous sulfate standard curve. Interpolated values were normalized to sample weight and expressed as μmol/L.

The TBARS assay provides a general assessment of lipid peroxidation products. Heart samples were combined with 4 mM butylated hydroxytoluene (W21,840‐5‐K; SAFC), 0.2 M phosphoric acid (26–36/37; RADCHEM), and 0.11 M TBA (STBB4876V; Sigma‐Aldrich) in a 1:0.125:1 ratio (modified from (Cairns et al., 2024)). The mixture was incubated at 90°C for 45 min to induce the reaction between MDA and TBA, followed by cooling on ice for 5 min. Butanol (BUT001; KIMIX) and a saturated salt solution (1.06404; Sigma‐Aldrich) were then added in a 2:0.5 ratio to the boiled samples to induce phase separation. After centrifugation at 10,000 ***g*** for 1 min (Spectrafuge 24D, Labnet International Inc., Edison, NJ, USA), the upper butanol layer containing the MDA‐TBA adduct was transferred to a clear 96‐well flat‐bottom plate. The absorbance was measured at 532 nm over 5 min, and the final MDA concentration was expressed as micromoles per liter (μmol/L).

### 2.6 Statistical analyses

All data was analyzed in GraphPad Prism v8.02 (GraphPad Software Inc., San Diego, California, USA). After normality testing (Shapiro–Wilk) and outlier detection (ROUT; *Q* = 1%), data was subjected to either a Student's *t*‐test or Mann–Whitney test to determine significance between control and CRS groups within each sex. For comparisons across more than two variables, three‐way ANOVAs with Fisher's LSD were performed where applicable. Any *p*‐value ≤0.05 was considered significant. Data is presented as mean ± standard deviation.

## 3 RESULTS

This study represents a molecular‐level extension of our previous work, which examined circulating biochemical and *ex vivo* heart functional parameters (Cairns et al., 2024). Here, male CRS rats exhibited attenuated circulating BDNF and testosterone levels, as well as low anxiety behavior with minimal alterations to HPA‐axis hormones. Conversely, CRS females showed altered HPA‐axis and sex hormone levels, as well as increased circulating cardiac troponin T and a global cellular senescence profile. While both sexes displayed relatively unchanged baseline cardiac function, CRS males and females exhibited greater functional recovery post‐ischemia, with the latter highlighting their sex‐specific vulnerability to ischemic injury in the context of chronic stress (Cairns et al., 2024).

High‐resolution respirometry of post‐reperfusion cardiac tissues revealed differences in complex activity and substrate utilization. Male CRS animals displayed elevated routine (*p* = 0.029) and β‐oxidation‐linked OXPHOS (*p* = 0.025) versus controls whereas CRS females displayed decreased ETF‐linked LEAK respiration (*p* = 0.011) relative to controls in non‐ischemic tissue (Table 1). Sex differences were also observed with control females displaying increased ETF‐linked LEAK respiration (*p* = 0.008) as well as RE (*p* = 0.026) and OPCE ratios (*p* = 0.010) compared to their male counterparts in non‐ischemic tissue (Table 1).

**TABLE 1:** The effects of chronic stress exposure on high resolution respirometry parameters in non‐ischemic and ischemia‐reperfused tissues of male and female rats subjected to *ex vivo* regional ischemia–reperfusion.

O_2_ flux (pmol·s^−1^·mg^−1^)	Male				Female			
	Non‐ischemic		Ischemia‐reperfused		Non‐ischemic		Ischemia‐reperfused	
	Control	CRS	Control	CRS	Control	CRS	Control	CRS
Routine	**3.03 ± 1.58**	**5.48 ± 2.89**	3.64 ± 1.93	3.78 ± 1.95	4.45 ± 1.33	3.78 ± 1.26	2.69 ± 0.68	3.59 ± 1.91
ETF‐LEAK	5.56 ± 1.64	6.41 ± 2.15	**10.58 ± 1.97**	**6.04 ± 2.69**	9.52 ± 4.39**	**5.85 ± 2.16**	4.39 ± 1.20****	4.84 ± 1.67
Β‐oxidation	**4.15 ± 1.54**	**8.64 ± 4.63**	7.34 ± 3.57	7.77 ± 4.89	5.46 ± 2.19	4.99 ± 2.35*	4.46 ± 3.84	3.25 ± 1.45*
Cytochrome C	6.87 ± 3.40	8.16 ± 2.31	8.54 ± 1.94	6.34 ± 2.60	7.01 ± 1.85	6.07 ± 3.44	4.34 ± 2.27**	3.45 ± 1.12
Complex I	7.02 ± 2.42	6.62 ± 2.02	9.15 ± 2.54	7.23 ± 1.76	7.88 ± 1.93	7.06 ± 1.75	4.97 ± 2.07*	3.45 ± 1.03**
Complex II	61.92 ± 17.43	55.34 ± 11.44	64.39 ± 14.26	57.96 ± 14.76	57.01 ± 8.73	61.13 ± 20.59	44.50 ± 17.85*	47.46 ± 13.55
ETS capacity	69.15 ± 18.63	62.18 ± 10.01	74.62 ± 16.06	68.60 ± 14.14	67.10 ± 10.24	74.48 ± 28.22	53.91 ± 16.64	54.54 ± 12.68
ROX	48.23 ± 13.72	44.23 ± 7.97	58.29 ± 12.86	47.05 ± 9.42	53.16 ± 9.87	56.04 ± 15.69	45.99 ± 12.62	40.32 ± 7.58
Complex IV	732.00 ± 234.300	741.10 ± 288.00	521.80 ± 72.01	741.30 ± 262.20	770.40 ± 400.60	548.60 ± 178.10	377.50 ± 102.9	608.20 ± 250.70

*Note*: Values represented as mean ± SD. Sample size: male (control = 6; CRS = 5); female (control = 5; CRS = 7). Data analyzed using a three‐way ANOVA with Fisher's LSD. Normality tested (Shapiro–Wilk) and outliers removed (ROUT, *Q* = 1%). Key: CI, complex I; CII, complex II; CIV, complex IV; ETF, electron‐transferring flavoprotein; ETS, electron‐transfer system; ROX, residual oxygen consumption. Significant differences between control and CRS groups in bold. *Symbols indicate significant differences between females and males of the sample experimental group. *p < 0.05; **p < 0.01. Shaded cells = significant differences between non‐ischemic and ischemia‐reperfused tissues within the experimental group (*p* < 0.05).

Ischemia‐reperfused tissue regions revealed further alterations in male and female CRS groups. Male CRS rats presented with decreased ETF‐linked LEAK respiration (*p* = 0.003) and OPCE (*p* = 0.035) ratio (Table 2), whereas the female CRS group displayed an attenuated RE ratio (*p* = 0.037) compared to their respective controls (Table 2). Female controls also showed lowered ETF‐linked LEAK (*p* < 0.0001), cytochrome c response (*p* = 0.009), complex I (*p* = 0.001) and complex II (*p* = 0.042) activity, and OPCE ratio (*p* = 0.020) in ischemia‐reperfused tissue compared to control males (Table 1). A decrease was observed in the CRS female group for β‐oxidation (3.25 ± 1.45 pmol·s^−1^·mg^−1^ vs. 7.77 ± 4.89 pmol·s^−1^·mg^−1^; *p* = 0.019) and complex I activity (3.45 ± 1.03 pmol·s^−1^·mg^−1^ vs. 7.23 ± 1.76 pmol·s^−1^·mg^−1^; *p* = 0.002) compared to their male counterparts (Table 1). Moreover, male control ischemia‐reperfused tissues presented with reduced RE (0.78 ± 0.06 pmol·s^−1^·mg^−1^ vs. 0.67 ± 0.10 pmol·s^−1^·mg^−1^; *p* = 0.029) and OPCE (0.15 ± 0.05 pmol·s^−1^·mg^−1^ vs. 0.07 ± 0.04 pmol·s^−1^·mg^−1^; *p* = 0.002) ratios relative to control non‐ischemic tissue, which was not reflected in the CRS group tissues.

**TABLE 2:** The effects of chronic stress exposure on high resolution respirometry control ratios of non‐ischemic and ischemia‐reperfused tissues of male and female rats subject to *ex vivo* regional ischemia–reperfusion.

O_2_ flux (pmol·s^−1^·mg^−1^)	Male				Female			
	Non‐ischemic		Ischemia‐reperfused		Non‐ischemic		Ischemia‐reperfused	
	Control	CRS	Control	CRS	Control	CRS	Control	CRS
Total PE ratio	0.96 ± 0.02	1.03 ± 0.07	0.97 ± 0.05	0.97 ± 0.17	0.93 ± 0.06	0.90 ± 0.08*	0.89 ± 0.15	0.92 ± 0.05
CII/ETS	0.90 ± 0.03	0.90 ± 0.04	0.86 ± 0.02	0.85 ± 0.12	0.85 ± 0.05	0.83 ± 0.06	0.80 ± 0.12	0.86 ± 0.07
RE ratio	0.67 ± 0.10	0.61 ± 0.14	0.78 ± 0.06	0.69 ± 0.05	0.79 ± 0.05*	0.78 ± 0.10**	**0.86 ± 0.03**	**0.75 ± 0.09**
OPCE	0.07 ± 0.04	0.09 ± 0.02	**0.15 ± 0.05**	**0.09 ± 0.05**	0.14 ± 0.05*	**0.09 ± 0.05**	0.09 ± 0.03*	0.09 ± 0.03

*Note*: Values represented as mean ± SD. Sample size: male (control = 6; CRS = 5); female (control = 5; CRS = 7). Data analyzed using a three‐way ANOVA with Fisher's LSD post‐hoc test. Normality tested (Shapiro–Wilk) and outliers removed (ROUT, *Q* = 1%). Key: CII, complex II; ETF, electron‐transferring flavoprotein; ETS, electron‐transfer system; OPCE, oxidative phosphorylation coupling efficiency; PE = (β‐oxidation linked OXOHOS + CII activity)/ETS; RE, ROX/ETS; ROX, residual oxygen consumption. Significant differences between control and CRS groups in bold. *Symbols indicate significant differences between females and males of the sample experimental group. *p < 0.05; **p < 0.01. Shaded cells = significant differences between non‐ischemic and ischemia‐reperfused tissues within the experimental group (*p* < 0.05).

**FIGURE 1:**
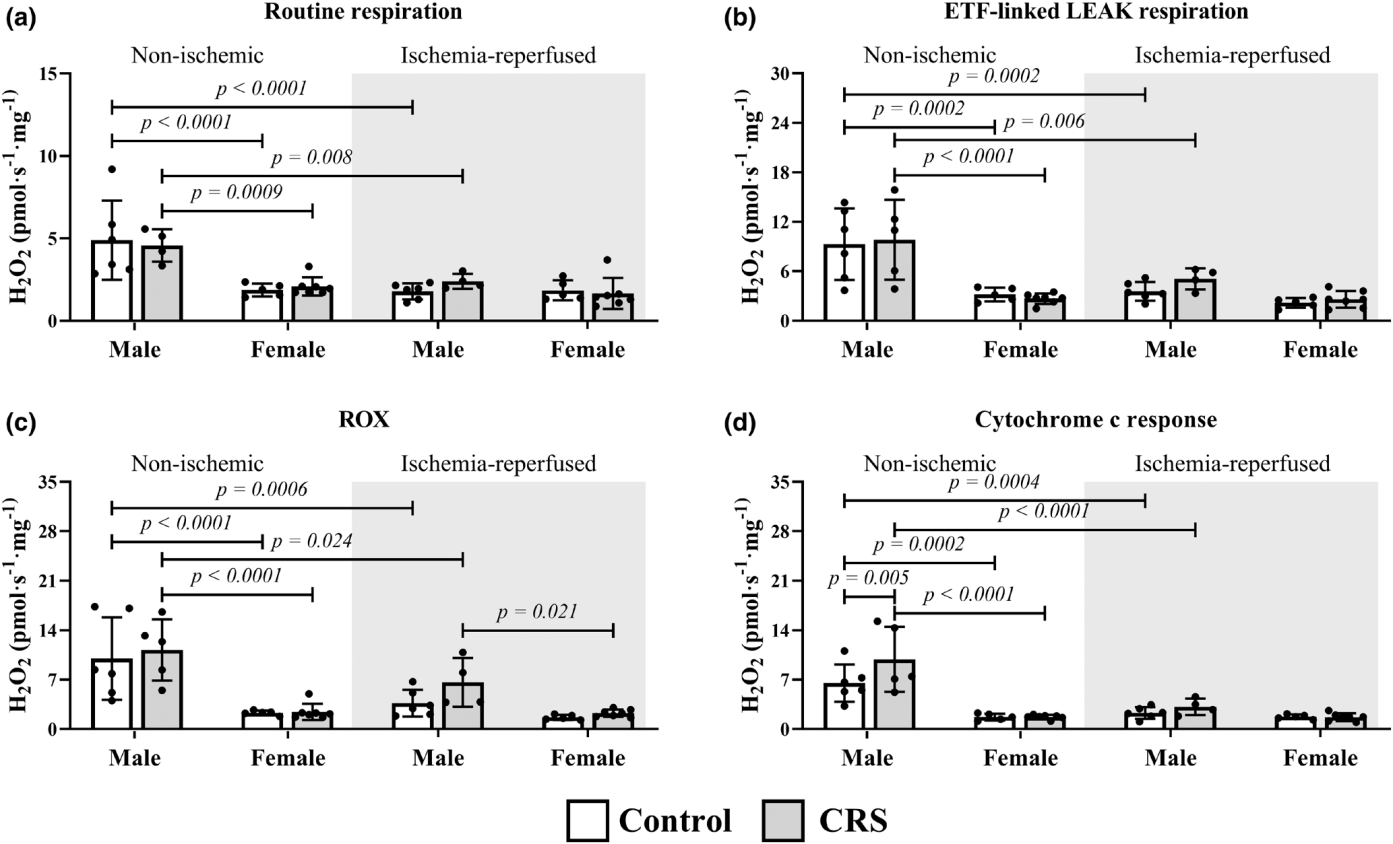
Females produced less hydrogen peroxide in post‐reperfusion non‐ischemic tissues. Key: White = control group; gray = CRS group; ETF, electron‐transferring flavoprotein‐linked non‐phosphorylating respiration; ROX, Residual oxygen consumption. Sample size: Male (*n* = 6 control; *n* = 5 CRS); female (*n* = 5 control; *n* = 7 CRS).

**FIGURE 2:**
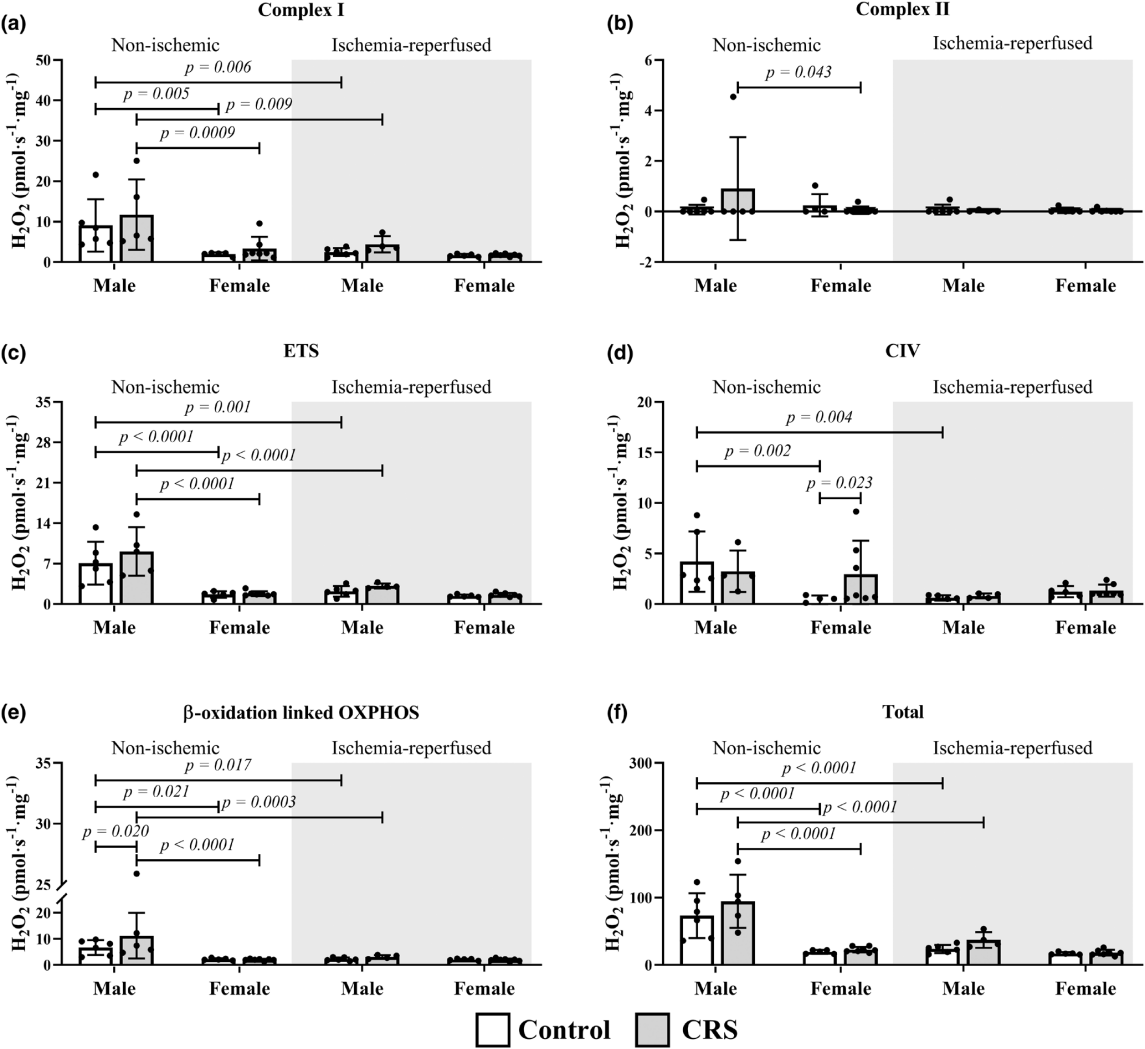
Females produced less hydrogen peroxide via complex activity in post‐reperfusion non‐ischemic tissues. Key: White = control group; gray = CRS group; CI, complex I; CII, complex II; ETS, electron‐transfer system; OXPHOS, oxidative phosphorylation. Sample size: Male (*n* = 6 control; *n* = 5 CRS); female (*n* = 5 control; *n* = 7 CRS).

There was a general trend for lower hydrogen peroxide production for the females versus males for several parameters in both non‐ischemic and ischemia‐reperfused tissues (Figure 1). In addition to increased non‐ischemic hydrogen peroxide levels in response to cytochrome c (9.88 ± 4.59 vs. 6.52 ± 2.61 pmol·s^−1^·mg^−1^; *p* = 0.005), male CRS rats produced elevated levels through β‐oxidation (11.23 ± 8.68 pmol·s^−1^·mg^−1^ vs. 6.71 ± 2.84 pmol·s^−1^·mg^−1^; *p* = 0.020) in non‐ischemic tissue relative to controls (Figure 2). Female CRS only displayed elevated complex IV‐associated hydrogen peroxide levels relative to controls (2.97 ± 3.30 pmol·s^−1^·mg^−1^ vs. 0.31 ± 0.54 pmol·s^−1^·mg^−1^; *p* = 0.023) in non‐ischemic tissue.

**FIGURE 3:**
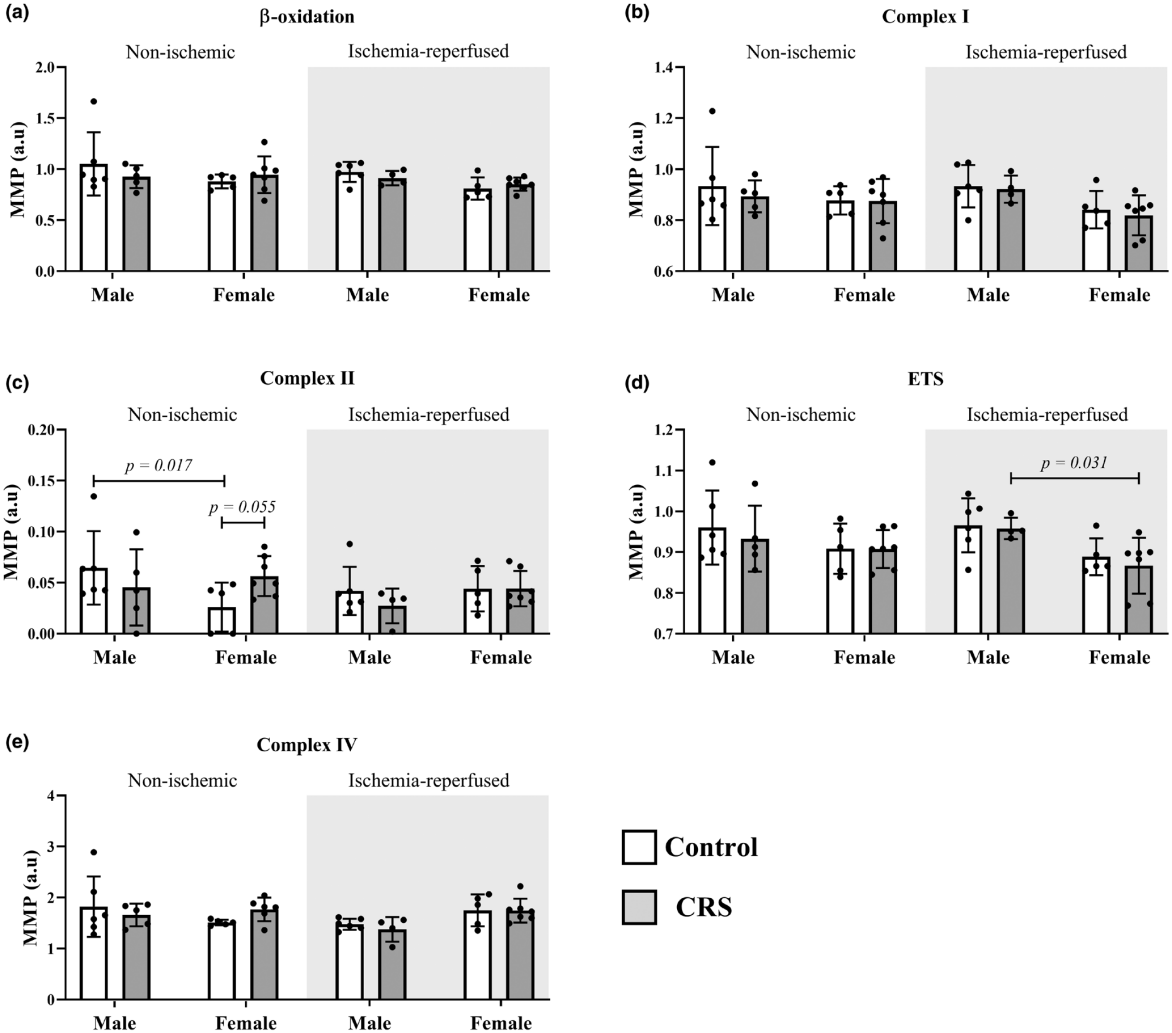
Chronic stress did not affect mitochondrial membrane potential in non‐ischemic and ischemia‐reperfused tissues. Key: White = control group; gray = CRS group; CI, complex I; CII, complex II; CIV, complex IV; ETS, electron‐transfer system. Sample size: Male (*n* = 6 control; *n* = 5 CRS); female (*n* = 5 control; *n* = 7 CRS).

Although control females displayed reduced complex II membrane potential compared to their male counterparts (0.03 ± 0.04 A.U. vs. 0.07 ± 0.04 A.U.; *p* = 0.017), this did not extend to the ischemia‐reperfused zone, nor between CRS groups (Figure 3).

**FIGURE 4:**
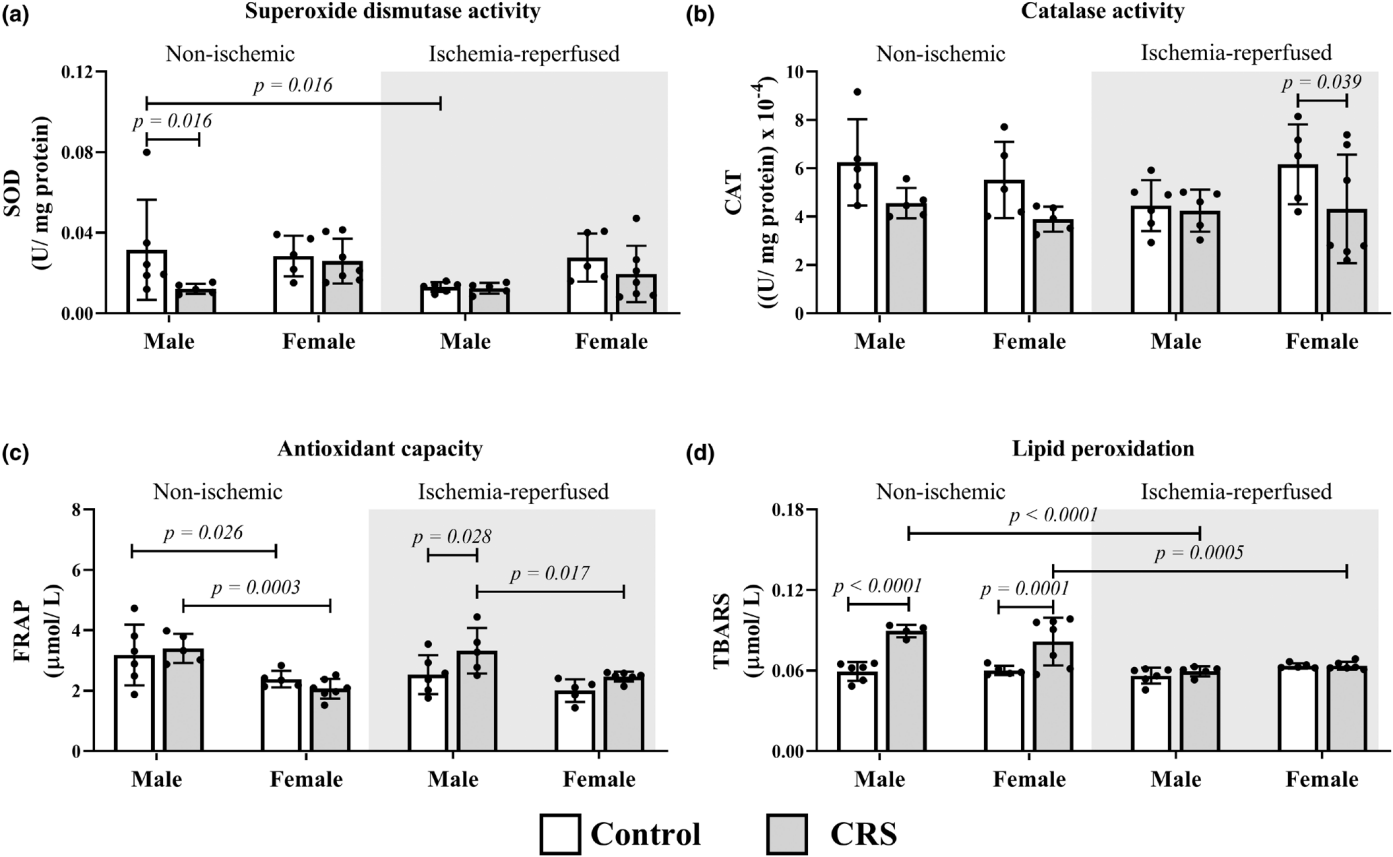
Chronic stress reduces male SOD activity in male non‐ischemic tissue but augments lipid peroxidation in both sexes. (a) SOD (b) CAT (male [*n* = 5 control non‐ischemic]); (c) FRAP (female [*n* = 5 control; *n* = 6 CRS]); (d) TBARS (female [*n* = 5 control; *n* = 6 CRS]). Sample size: Male (*n* = 6 control; *n* = 5 CRS) and female (*n* = 5 control, *n* = 7 CRS) across non‐ischemic and ischemia‐reperfused tissue regions unless specified. Key: White = control group; gray = CRS group.

Male CRS animals displayed reduced SOD activity (0.012 ± 0.003 U/mg protein vs. 0.032 ± 0.025 U/mg protein; *p* = 0.017) and elevated lipid peroxidation (0.082 ± 0.016 μmol/L vs. 0.059 ± 0.007 μmol/L; *p* = 0.009) in non‐ischemic tissue with elevated FRAP in ischemia‐reperfused regions (3.322 ± 0.755 μmol/L vs. 2.533 ± 0.642 μmol/L; *p* = 0.024) relative to controls (Figure 4). Like their male counterparts, female CRS animals also presented with elevated lipid peroxidation (0.082 ± 0.018 μmol/L vs. 0.060 ± 0.004 μmol/L; *p* = 0.025) in non‐ischemic regions in combination with increased ischemia‐reperfused FRAP (2.465 ± 0.163 μmol/L vs. 2.006 ± 0.373 μmol/L; *p* = 0.017) relative to their controls (Figure 4).

## 4 DISCUSSION

Preclinical studies established that chronic stress is a significant contributor to systemic pathology, with a potential sex‐based disparity in susceptibility to myocardial ischemic reperfusion injury. Here, chronically stressed male subjects largely exhibited greater infarct sizes and diminished cardiac functional recovery compared to females, indicating a higher vulnerability (Lagranha et al., 2010; Penna et al., 2009; Scott et al., 2022). However, our previous *ex vivo* preclinical research demonstrated attenuated function in both male and female hearts, with females displaying a greater functional deficit following reperfusion (Cairns et al., 2024). Whether such attenuated functional recovery is a consequence of impaired mitochondrial function or redox imbalance required further investigation.

This study therefore aimed to characterize the effects of chronic stress on cardiac mitochondrial respiratory function in ischemia‐reperfused and non‐ischemic cardiac tissues and in a sex‐specific manner. Our results demonstrate a shift towards cardiac fatty acid metabolism in male non‐ischemic tissues with significant lipid peroxidation. Conversely, females displayed minimal changes in both oxygen consumption and free radical production across ischemic zones, although we observed increased membrane potential and ATP levels in non‐ischemic CRS hearts (see Figure S3). Moreover, oxidative stress assays revealed alterations to antioxidant defenses across ischemic zones in both male and female CRS animals with increased oxidative damage in non‐ischemic tissues. Our data indicate that sex‐based differences and ischemia‐reperfusion injury are the primary determinants of altered mitochondrial respiration, with chronic stress exerting a less significant influence.

While fresh samples are considered the gold standard for high‐resolution respirometry, several studies found that frozen samples, even without cryopreservation, can still be utilized to assess mitochondrial function (Acin‐Perez et al., 2020; Sarver et al., 2024; Yao et al., 2023). This is particularly relevant for long‐term studies where sample storage is often essential. In support, Acin‐Perez et al. (2020) confirmed that mitochondrial respiration can be assessed in freeze‐thawed samples and that it reflected the different experimental alterations. Although ATP‐coupled respiration and substrate shuttle carriers were negatively affected, electron transfer capacity remained intact in the frozen samples. In our study, the low ADP response observed following titration can be attributed to ineffective permeabilization or a loss of coupling due to the freezing process. Despite the potential negative effects of freezing, our results indicate that mitochondria in such samples remained functionally coupled. This is evident from the complex II‐linked OXPHOS/ETS capacity and total PE ratios, which demonstrate that a significantly greater proportion of oxygen is utilized for ATP production compared to non‐ATP side reactions or proton leak.

### 4.1 Effects of ischemia

Direct comparisons between ischemia–reperfusion studies are difficult as the methodological variations (*in vivo* versus *ex vivo* models) and types of perfusate used, as well as the duration/induction of ischemia, can all influence the tissue microenvironment prior to sample collection. Here, adult male Sprague–Dawley rats subjected to global ischemia (without reperfusion) displayed greater mitochondrial damage and loss of function *ex vivo* (Quader et al., 2020). Of note, comparisons within the *ex vivo* setting can prove challenging as such models often differ in ischemia duration, reperfusion time, pacing, and mitochondrial isolation methods (Lishmanov et al., 2014; Venditti et al., 2001). However, despite such challenges mitochondrial studies employing *ex vivo* ischemia‐reperfused heart tissues consistently showed lowered State 3 (ADP‐stimulated) oxygen consumption compared to controls, while State 2 (malate and glutamate) and State 4 (ATP synthesis) respiration remained unaffected (Lishmanov et al., 2014; Venditti et al., 2001).

Eickelmann et al. (2023) extended such findings to an *in vivo* porcine model of regional ischemia, observing attenuated State 3 respiration in ischemia‐reperfused tissues without changes in baseline respiration or complex IV activity compared to remote, non‐ischemic tissues. Our data reflects these findings to some extent, with no alterations in baseline respiration or complex IV activity in male control samples, while ETF‐linked LEAK respiration was elevated in the ischemia‐reperfused tissues. For the female controls, the ischemia‐reperfused tissues exhibited diminished ETF‐linked LEAK respiration, complex I, and complex IV respiration relative to non‐ischemic tissues, indicating lower basal aerobic respiration.

### 4.2 Male versus female

A recent meta‐analysis reported higher mitochondrial content in females, yet no differences in OXPHOS protein abundance or activity (Junker et al., 2022). Consistent with previous studies, male and female control animals did not differ for the majority of mitochondrial parameters measured within the non‐ischemic tissues, with the exception of ETF‐linked LEAK respiration (Chen et al., 2023). The ETF transfers electrons from flavoenzymes into the electron transfer chain in order to maintain the proton gradient across the inner mitochondrial membrane in the absence of ATP synthesis (reviewed by Henriques et al. (2021)). Shunting oxygen towards maintaining the proton motive force as opposed to ETS, ATP production decreases the energy output of mitochondria and increases oxidative stress (Simard et al., 2018). Yet the female controls did not display greater oxidative damage relative to males, likely as a result of the antioxidant effects of estrogen (Tsai et al., 2012). In support, supplementing ovariectomized mice with 17‐β‐estradiol decreased membrane microviscosity and improved function independent of mitochondrial content in skeletal muscle (Torres et al., 2018). Moreover, others found that females presented with an overall decrease in hydrogen peroxide production, suggesting some protection against oxidative stress (Chen et al., 2023). The vast majority of literature reports on a greater antioxidant capacity in females combined with attenuated hydrogen peroxide production, which was reflected in the non‐ischemic tissues of our study (Colom et al., 2007; Lagranha et al., 2010; Malorni et al., 2007). We propose that one potential mechanism may involve increased aldehyde dehydrogenase‐2 activity in female control hearts that can attenuate ROS generation to decrease cardiac injury (Lagranha et al., 2010).

### 4.3 Male effects

Increased β‐oxidation‐linked OXPHOS in the non‐ischemic tissues of male CRS animals, coupled with the elevated PE ratio, suggest mitochondrial compensatory adaptation to the ischemic insult to ensure sustained contractility and function. It is well‐established that hearts increase β‐oxidation‐linked OXPHOS in response to ischemia to increase intracellular energy supply (Fillmore et al., 2014). However, this can become maladaptive due to oxygen wastage and pro‐oxidant production under ischemic conditions as previously highlighted by us (Essop & Opie, 2004, 2020). The increased reliance on β‐oxidation 2 h into reperfusion suggests a predisposition for long‐term cardiac damage in males, yet whether this is due to increased protein activity and/or expression of modulators in such pathways warrants further investigation. Thus, we propose that for some non‐ischemic parts of the heart, the relatively higher fatty acid β‐oxidation will contribute to damage through increased ROS production and cell death if sustained (Essop & Opie, 2004, 2020).

While high‐resolution respirometry data offers insights into specific complex activities, routine respiration more directly reflects the metabolic state of samples undergoing oxidative stress assessments. Although this respiratory state was increased in non‐ischemic tissues of CRS males, there was not a concurrent increase in hydrogen peroxide levels. Superoxide dismutase activity is influenced by several factors, but perhaps the most applicable in this context is testosterone—more specifically attenuated circulatory levels in CRS males. Previous studies on castrated rats or testicular feminized mice reported attenuated myocardial SOD activity through androgen receptor‐independent mechanisms (Zhang et al., 2011, 2013). Our results support this dynamic with lowered non‐ischemic SOD activity. We suggest that decreased SOD activity results in a decreased conversion of superoxide radicals to hydrogen peroxide. This in turn will lead to lower levels of hydrogen peroxide for CAT to scavenge. It is our opinion that the comparable CAT activity likely represents the diminished supply of SOD‐related hydrogen peroxide. This together with the increased TBARS in non‐ischemic CRS male tissues indicates that superoxide may be the primary mediator of myocardial damage (Ighodaro & Akinloye, 2018). For example, peroxynitrite can directly modify tyrosine residues of contractile proteins thereby decreasing calcium sensitivity and impairing contraction (Pérez de la Lastra et al., 2022; Supinski et al., 1999).

Of note, the observed changes in mitochondrial respiration were recorded in well‐perfused regions, where compensatory adaptations may enhance metabolic activity in the non‐ischemic myocardium. While previous studies documented a decline in mitochondrial respiration in ischemia‐reperfused regions, our data indicate minimal alterations (Table S1), potentially due to the freezing process masking specific regional variations (Eickelmann et al., 2023). Moreover, the increased FRAP in the ischemia‐reperfused myocardium together with relatively stable SOD and CAT activities suggest the involvement of non‐enzymatic endogenous antioxidant defense systems such as glutathione to effectively neutralize ROS generated during ischemia (Matuz‐Mares et al., 2021). While our findings diverge from some established studies, they underscore the necessity of considering regional metabolic adaptations and methodological factors when interpreting mitochondrial function in the ischemia‐reperfused and non‐ischemic myocardium.

### 4.4 Female effects

Cortisol and estradiol are both anti‐inflammatory agents, with the latter considered cardioprotective (Hou et al., 2021; Lin et al., 2018; Pasieka & Rafacho, 2016). The significant decrease in such hormones prior to *ex vivo* perfusions and regional ischemia suggests an underlying degree of inflammation as we stated previously (Cairns et al., 2024). In support, our previous work reported elevated tumor necrosis factor‐alpha levels in CRS female serum together with increased circulating cardiac troponin T levels (Cairns et al., 2024). Thus, increased overt free radical production with substantial metabolic alterations would be predicted after regional ischemia–reperfusion. Our redox data supports significantly elevated lipid peroxidation in non‐ischemic tissues yet did not extend to impaired antioxidant defenses. As with the male tissues, the influence of reactive nitrogen species remains unexplored and may provide additional mechanistic insight.

As only ETF‐linked LEAK respiration was attenuated in this region, females displayed a relatively enhanced mitochondrial tolerance to both chronic stress and ischemia–reperfusion. A decrease in ETF‐linked LEAK respiration suggests that less oxygen was utilized to maintain the proton motive force and that the ETS was outperforming the controls. Moreover, increased ATP levels (see Figure S3) in non‐ischemic tissues suggest that while substantial oxidative damage was present in this region, the chronic stress protocol did not impair OXPHOS energy production in the females. Previous unpublished work by our laboratory supports this with an increase in ATP synthase and fusion/fission ratio in frozen CRS female cardiac tissues (in the absence of *ex vivo* perfusions).

Like males, CRS females displayed an attenuated RE ratio compared to controls in ischemia‐reperfused regions, leading to lower non‐ETS side reactions and improved mitochondrial coupling. However, the relatively modest decrease in ATP production (Figure S3) in ischemia‐reperfused regions suggests that improved OXPHOS coupling didn't necessarily translate to increased energy production. Together these data indicate that the decreased functional recovery we observed in stressed females is likely not mediated via mitochondrial effects. The moderate decrease in ATP levels may play a role, but we propose that lowered cardiac function in the stressed females likely occurs due to alterations in the contractile apparatus of the heart. The lack of lipid peroxidation in the CRS female ischemia‐reperfused tissues was surprising as previous studies found increased oxidative damage following ischemic insult (Wei et al., 2017).

## 5 CONCLUSION

Our findings highlight significant differences in mitochondrial adaptations and oxidative stress responses between male and female rats subjected to CRS and subsequent ischemia–reperfusion. Males exhibit a compensatory increase in fatty β‐oxidation OXPHOS in non‐ischemic tissues, which may predispose them to long‐term cardiac damage due to heightened oxidative stress mediated by superoxide. While females exhibited enhanced mitochondrial coupling in non‐ischemic tissues, the mitochondrial data collectively suggest that alternative mechanisms (vs. mitochondrial) are responsible for decreased cardiac function observed in this instance. These insights underscore the importance of considering sex differences in cardiac responses to stress and ischemic injury, suggesting that targeted therapeutic strategies may be necessary to address the unique vulnerabilities and protective mechanisms present in each sex.

### 5.1 Limitations

As discussed previously, although frozen tissues are an acceptable sample type for mitochondrial respiratory studies, the sample preparation needs careful consideration. For example, isolated mitochondria, permeabilized muscle fibers, and tissue homogenates (derived from frozen samples) do require additional considerations for oxygen consumption measurements in order to match the outcomes expected when using fresh tissue preparations (Acin‐Perez et al., 2020). Permeabilized fibers also require mechanical and chemical disruption, as well as hyperoxic conditions, which may interfere with ROS production (Gnaiger, 2001). Thus, we recommend that future studies utilizing frozen tissues to investigate both oxygen consumption and ROS production should consider using either isolated mitochondria or tissue homogenates.

## AUTHOR CONTRIBUTIONS

Megan Cairns, Jasmine Andrews, Caitlin Paige Odendaal, Cassidy O'Brien, Balindiwe Sishi, and M. Faadiel Essop designed the study. Megan Cairns, Erna Marais, and Fanie Rautenbach performed the experiments. Megan Cairns, Gerald Maarman, Fanie Rautenbach, Danzil Joseph, Jeanine Marnewick, and M. Faadiel Essop analyzed the data and prepared the figures. Megan Cairns, Gerald Maarman, and M. Faadiel Essop drafted, edited, and revised the manuscript. All authors have read and approved the final version of the manuscript.

## FUNDING INFORMATION

The authors wish to acknowledge funding provided by the National Research Foundation (NRF) of South Africa to M. F. Essop (grant numbers: 129325 and CPRR230508103517).

## CONFLICT OF INTEREST STATEMENT

The authors confirm that there is no conflict of interest.

## Supporting information


Appendix S1.

## Data Availability

The data that support the findings of this study are available on request from the corresponding author and will be openly available in a repository when the article is accepted for publication.
