# Supplementary material for: An investigation into the sex dependence of post‐reperfusion cardiac mitochondrial function and redox balance in chronically stressed rats
Source: Physiol Rep. 2025 Feb 25;13(5):e70185. doi: 10.14814/phy2.70185 (PMC11859663; doi:10.14814/phy2.70185)
Supplement: Supplementary file 1 — Appendix S1. [file PHY2-13-e70185-s001.docx]

***Supplemental Table 1: The substrate-inhibitor titrations protocol employed using the Oroboros O2k respirometer according to the SUIT-005_O2_pfi_D011 protocol (in order of addition).***

| **Agent** | **Volume (μL)** | **[Final] (mM)** | **Agent type** | **Site of action** | **Catalog number** |
| --- | --- | --- | --- | --- | --- |
| Octanoylcarnitine | 10 | 0.5 | Substrate | ETF | O6206 |
| Malate # | 10 | 2 | Substrate | CI | M1000 |
| ADP | 30 | 7.5 | Substrate | ATP synthase | 177105 |
| Cytochrome c | 5 | 10 | Substrate | CIV | C7752 |
| Pyruvate | 5 | 5 | Substrate | CI | S2378 |
| Succinate | 20 | 10 | Substrate | CII | S2378 |
| CCCP | 1 | 0.5 | Uncoupler | $\Delta\mu$_H+_ | C2759 |
| Rotenone | 1 | 0.5 | Inhibitor (CI) | CI | R8875 |
| Antimycin-A | 1 | 2.5 | Inhibitor (CIII) | CIII | A8674 |
| Ascorbate | 5 | 2 | Substrate | CIV | A7631 |
| TMPD # | 5 | 0.5 | Substrate | CIV | T3134 |
| Sodium azide | 100 | 200 | Inhibitor (CIV) | CIV | S2002 |

Key# – titrated directly after the previous agent without O_2_ consumption line stabilization; ETF – electron transfer flavoprotein; CI – complex I; CII – complex II; CIII – complex III; CIV – complex IV; $\Delta\mu$H+ – proton gradient.


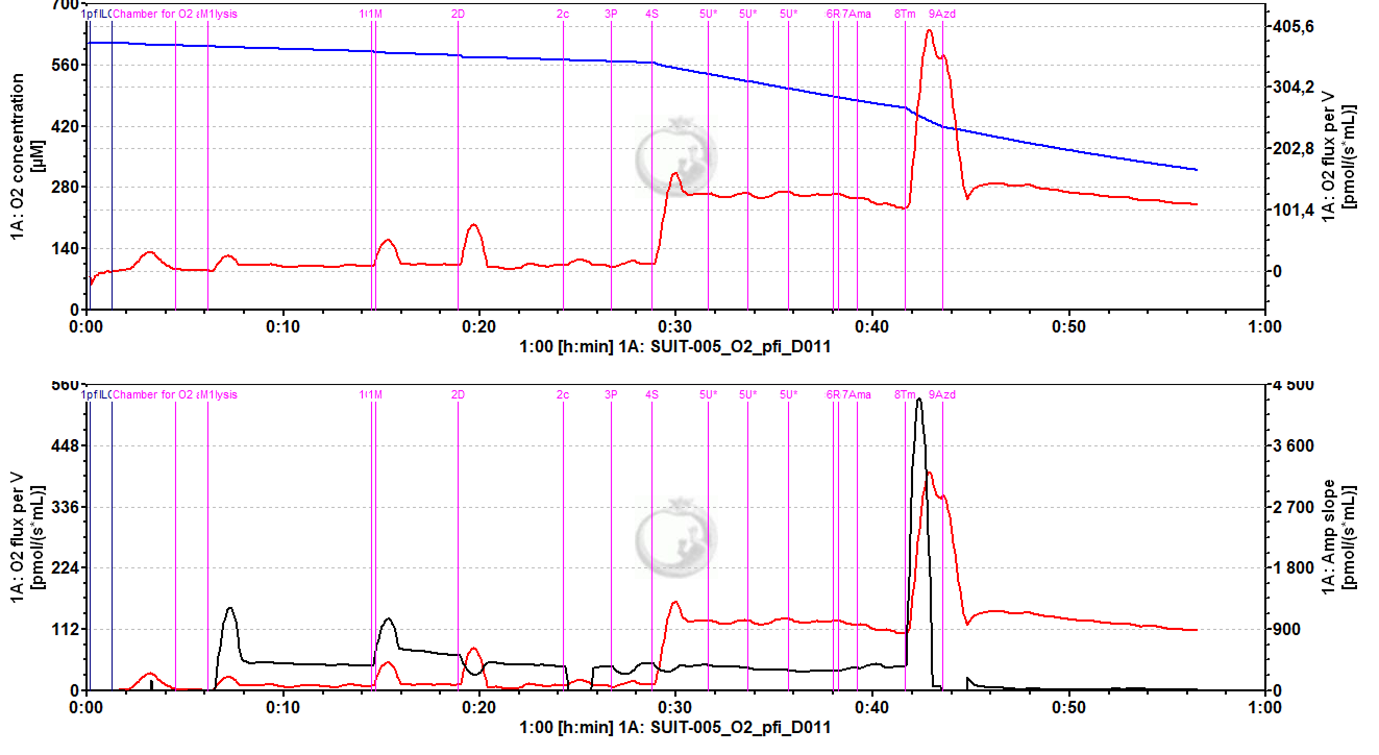


**Supplemental figure 1:** **Representative DatLab oxygraphs of frozen, ischemia-reperfused heart tissues subjected to the SUIT-005_O2_pfi_D011 protocol. Key:** Red line = O_2_ consumption (pmol/ (s*mL)); blue line = O_2_ concentration; black line = resorufin fluorescent signal (pmol/ (s*mL)); vertical purple lines = titration steps.


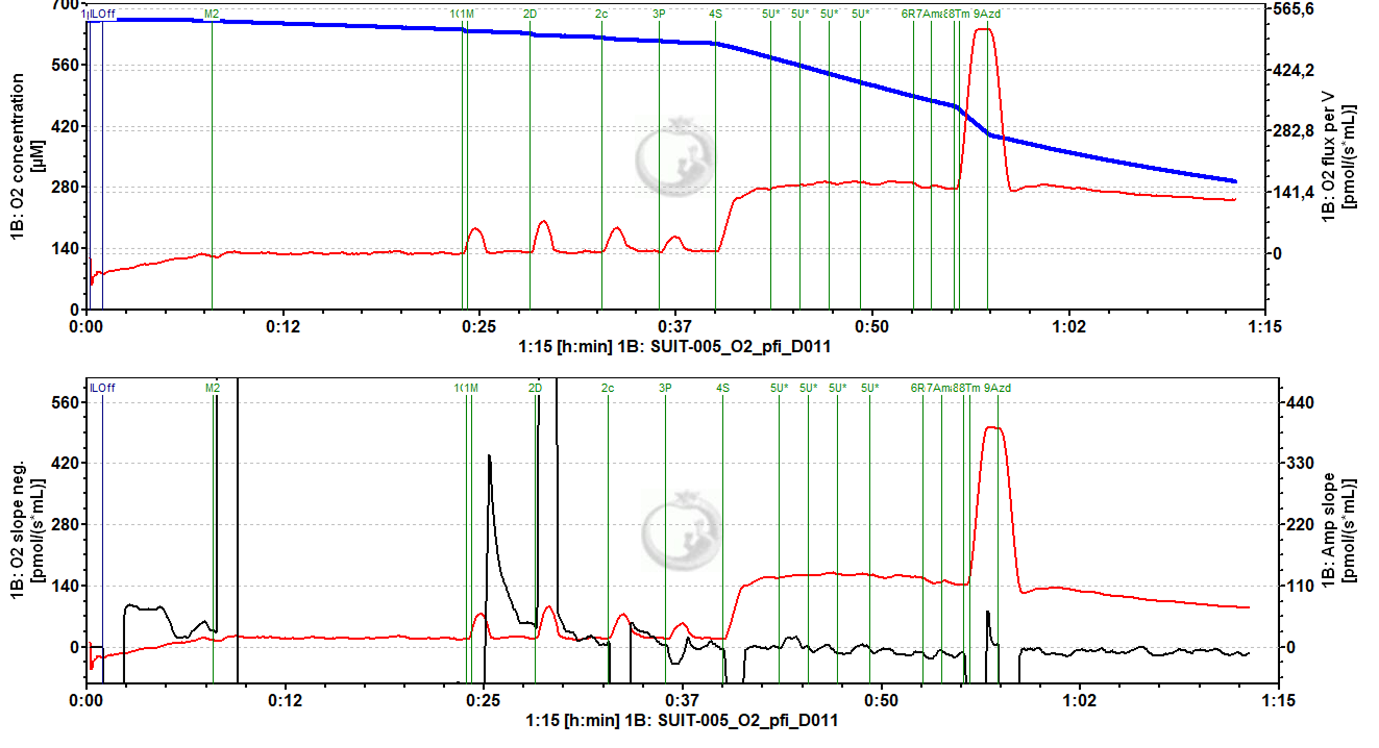


**Supplemental figure 2:** **Representative DatLab oxygraphs of frozen, ischemia-reperfused heart tissues subjected to the SUIT-005_O2_pfi_D011 protocol. Key:** Red line = O_2_ consumption (pmol/ (s*mL)); blue line = O_2_ concentration; black line = TMRM fluorescent signal (pmol/ (s*mL)); vertical purple lines = titration steps.


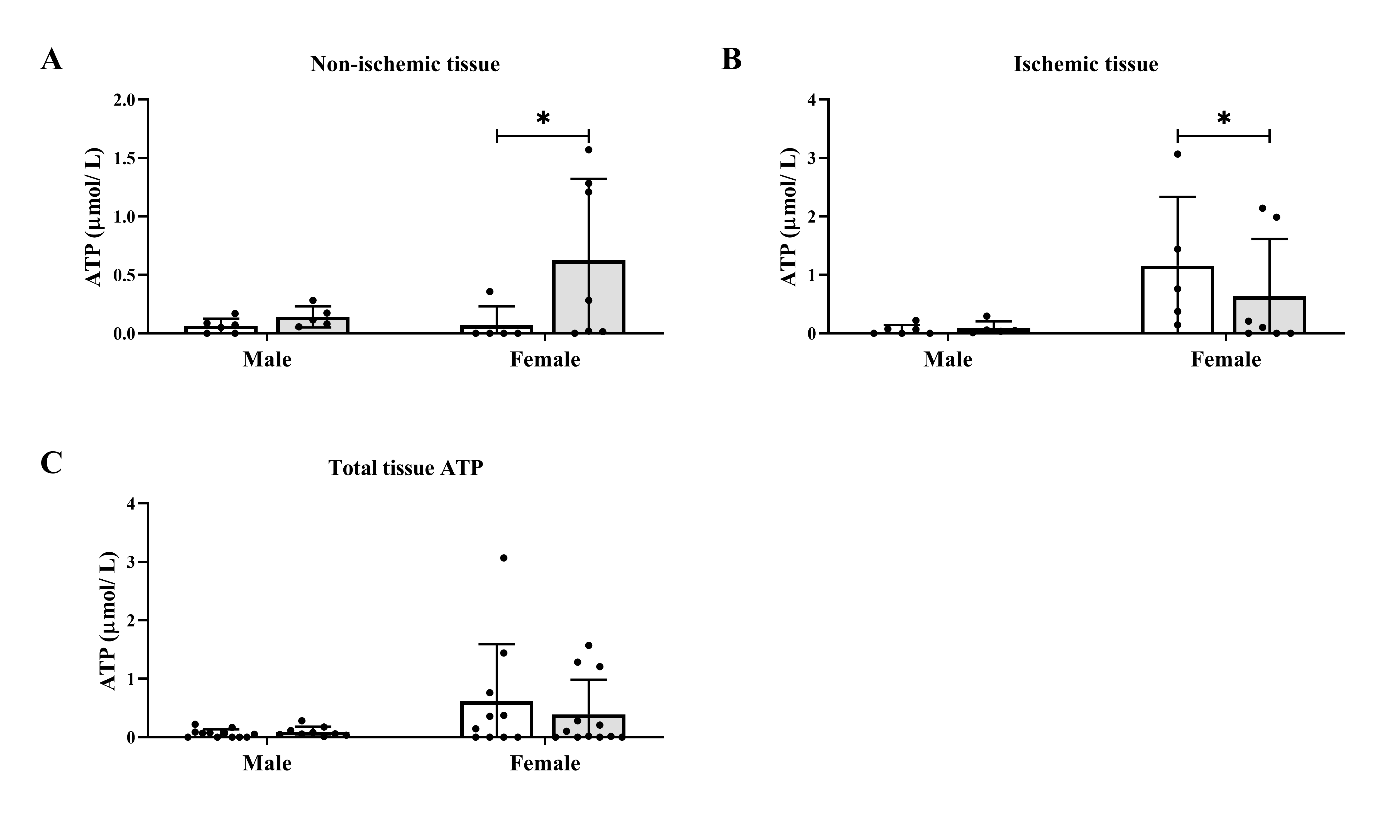


***Supplemental figure 3:*** ***ATP determination on frozen, ischemia-reperfused heart samples.*** *Although CRS females displayed elevated and reduced ATP levels verses controls in non-ischemic and ischemic tissue respectively, there is a large degree of variation and very low abundance levels across all samples. Samples were stored at -80°C for ~2 years post ex vivo perfusion, and ~1 year post high-resolution respirometry measurements, before ATP determination.* ***Key:*** *white bars = control; grey bars = CRS. * p < 0.05.* *Data presented as mean ± SD.*
